# Infections with *Plasmodium falciparum* during pregnancy affect VAR2CSA DBL-5 domain-specific T cell cytokine responses

**DOI:** 10.1186/s12936-016-1525-x

**Published:** 2016-09-21

**Authors:** Komi Gbédandé, Gilles Cottrell, Bertin Vianou, Samad Ibitokou, Aurax Fernando, Marita Troye-Blomberg, Ali Salanti, Kabirou Moutairou, Achille Massougbodji, Nicaise Tuikue Ndam, Philippe Deloron, Adrian J. F. Luty, Nadine Fievet

**Affiliations:** 1 Centre d’Etude et de Recherche sur le Paludisme Associé à la Grossesse et à l’Enfance (CERPAGE), Faculté des Sciences de la Santé, Université d’Abomey-Calavi, Cotonou, Benin; 2 Département de Biochimie et de Biologie Cellulaire, Faculté des Sciences et Techniques, Université d’Abomey-Calavi, Cotonou, Benin; 3 Institut de Recherche pour le Développement, MERIT UMR D216 Mère et enfant face aux infections tropicales, Paris, France; 4 COMUE Sorbonne Paris Cité, Faculté de Pharmacie, Université Paris Descartes, Paris, France; 5 Department of Molecular Biosciences, the Wenner-Gren Institute, Stockholm University, Stockholm, Sweden; 6 Department of International Health, Institute of International Health, Immunology and Microbiology, University of Copenhagen, Copenhagen, Denmark

**Keywords:** Malaria, Pregnancy, VAR2CSA, Cytokines, T cells

## Abstract

**Background:**

Current knowledge of human immunological responses to pregnancy-associated malaria-specific *Plasmodium falciparum* protein VAR2CSA concerns almost exclusively B cell-driven antibody-mediated activity. Knowledge of VAR2CSA-specific T cell-mediated activity is minimal by comparison, with only a single published report of a study investigating VAR2CSA-derived peptide-specific T cell responses. The study described here represents an attempt to redress this balance.

**Methods:**

Within the framework of a cohort study of 1037 pregnant Beninese, sub-groups were selected on the basis of the documented presence/absence of infection with *P. falciparum* and conducted detailed immunological assessments both at inclusion into the study and at delivery. Peripheral blood mononuclear cells were isolated, stimulated in vitro, and VAR2CSA DBL-5 domain-specific, IFN-γ-secreting T-cell frequencies and cytokine responses were quantified using flow cytometric techniques. Multivariate analyses were used to determine primarily whether the T cell-mediated DBL5-specific activity measured was associated with infection by *P. falciparum* adjusted for gravidity, anaemia and other cofactors.

**Results:**

Infections with *P. falciparum* detected at inclusion were associated with enhanced non-specific TNF responses, whilst diminished non-specific and DBL-5-specific IL-10 responses were associated with infections detected at delivery. Infections during pregnancy led to enhanced non-specific and DBL-5-specific IFN-γ responses detectable at delivery but to concomitantly lower DBL-5-specific CD8^+^ IFN-γ responses. Prospective assessments indicated that non-specific pro-inflammatory responses detectable at inclusion in the study were associated with the occurrence of infections subsequently during pregnancy.

**Conclusions:**

The findings represent a first step in elucidating the quantity and quality of cellular immunological responses to VAR2CSA, which will help in the development of the primary vaccine candidate for prevention of pregnancy-associated malaria.

**Electronic supplementary material:**

The online version of this article (doi:10.1186/s12936-016-1525-x) contains supplementary material, which is available to authorized users.

## Background

Pregnancy-associated malaria (PAM) due to infection with *Plasmodium falciparum* is a well-recognized, preventable cause of morbidity and mortality that impacts maternal, neonatal and infant health [1, 2]. Prevention of PAM currently relies on the distribution of long-lasting insecticide-impregnated nets (LLINs) and use of the anti-malarial drug combination sulfadoxine-pyrimethamine (SP) for intermittent preventive treatment during pregnancy (IPTp). The World Health Organization (WHO) now recommends SP-IPTp given at least three times with monthly spacing during pregnancy, beginning in the second trimester. Both those existing tools suffer from significant insufficiencies in distribution and uptake as well as from the fact that resistance of *Anopheles* mosquitoes to insecticides and of *P. falciparum* parasites to SP, is now widespread across sub-Saharan Africa [3]. A vaccine to prevent PAM would thus represent a valuable addition to the current set of tools available.

The study described here formed part of the EU FP7-funded STOPPAM project (strategies to prevent pregnancy-associated malaria). STOPPAM aimed to generate the information necessary to accelerate the development of a vaccine to prevent PAM by conducting in-depth longitudinal studies of large numbers of pregnant women in Benin and Tanzania. Malaria infection may occur any time during the 9 months of pregnancy. The STOPPAM cohort enabled to demonstrate that early infection during pregnancy is associated with higher risk of low birth weight and anemia at delivery [4, 5]. The results of those studies pointed strongly towards the existence of a naturally acquired antibody-mediated form of immunity that rendered multigravidae women significantly less at risk of PAM than first-time mothers [6], with the consensus that the primary target of the antibodies induced is a parasite-derived protein called VAR2CSA. Antibodies prevent the binding of infected erythrocytes, via the VAR2CSA expressed on their surface membranes, to their specific placental receptor, namely chondroitin sulphate A (CSA) expressed by syncytiotrophoblast [7, 8]. A wealth of information has been generated on multiple aspects of VAR2CSA-specific antibodies and the B cells responsible for their production [9–11]. Typically, VAR2CSA-specific IgG responses have been shown to display a strong gender- and gravidity-specific profile, pointing to the acquisition of protection against PAM during successive pregnancies [12–14]. Similar information on T cell-specific response to VAR2CSA is virtually non-existent.

The availability of such information could clearly be a valuable adjunct in the context of ongoing efforts to develop a vaccine to prevent PAM, since immunological memory requires the induction of antigen-specific T-cell cytokine-driven help for the efficient generation of B cells producing isotype-switched, affinity-matured, functionally active antibodies [15–17]. The VAR2CSA-DBL-5 domain displays highly strain-transcendent epitopes suggesting a role for additive or synergistic vaccine strategies that would combine both broad adhesion-blocking and opsonizing antibody responses to prevent high-density placental infections associated with disease [18].

The aim of the study described here was to investigate the T-cell responses to the DBL-5 domain (in 2008 it was considered one of the leading such candidates) in a cohort of pregnant women naturally exposed to malaria during their pregnancies. The authors’ hypothesis was that infections occurring during pregnancy versus infections occurring only at delivery would have differential impacts on immune responses. Within the well-documented STOPPAM cohort, the study focused on two specific sub-groups in order to investigate: (i) cellular immunological responses early in pregnancy as a predictor of *P. falciparum* infections during pregnancy; (ii) consequences of malaria and anaemia events during pregnancy and at delivery on immune responses at delivery.

## Methods

### Study design

The STOPPAM project ‘strategies to prevent pregnancy associated malaria’ was conducted in Benin and Tanzania between November 2008 and April 2011. In Benin, the study took place in the district of Come, Mono Province, located 70 km west of the economic capital, Cotonou. Malaria transmission in the area has two peaks during the rainy seasons (from April to July, and from September to November). The entomological inoculation rate is 35–60 infective bites per person per year [19]. The STOPPAM study design has been described in detail elsewhere [5]. Some 1037 women under 24 weeks of gestation were enrolled in three antenatal clinics: Come, Akodeha and Ouedeme Pedah. They were followed monthly with clinical and parasitological surveillance up to and including delivery. According to national policy at the time of the study, pregnant women received IPTp with SP during their scheduled antenatal clinic visits (ANV). Women diagnosed with a clinical malaria attack (axillary temperature ≥37.5 °C with a positive malaria rapid diagnostic test (RDT) and/or a positive TBS) received a full treatment course of quinine. In the case of clinical symptoms between ANV, mothers were encouraged to attend the maternity clinic to receive care.

**Fig. 1:**
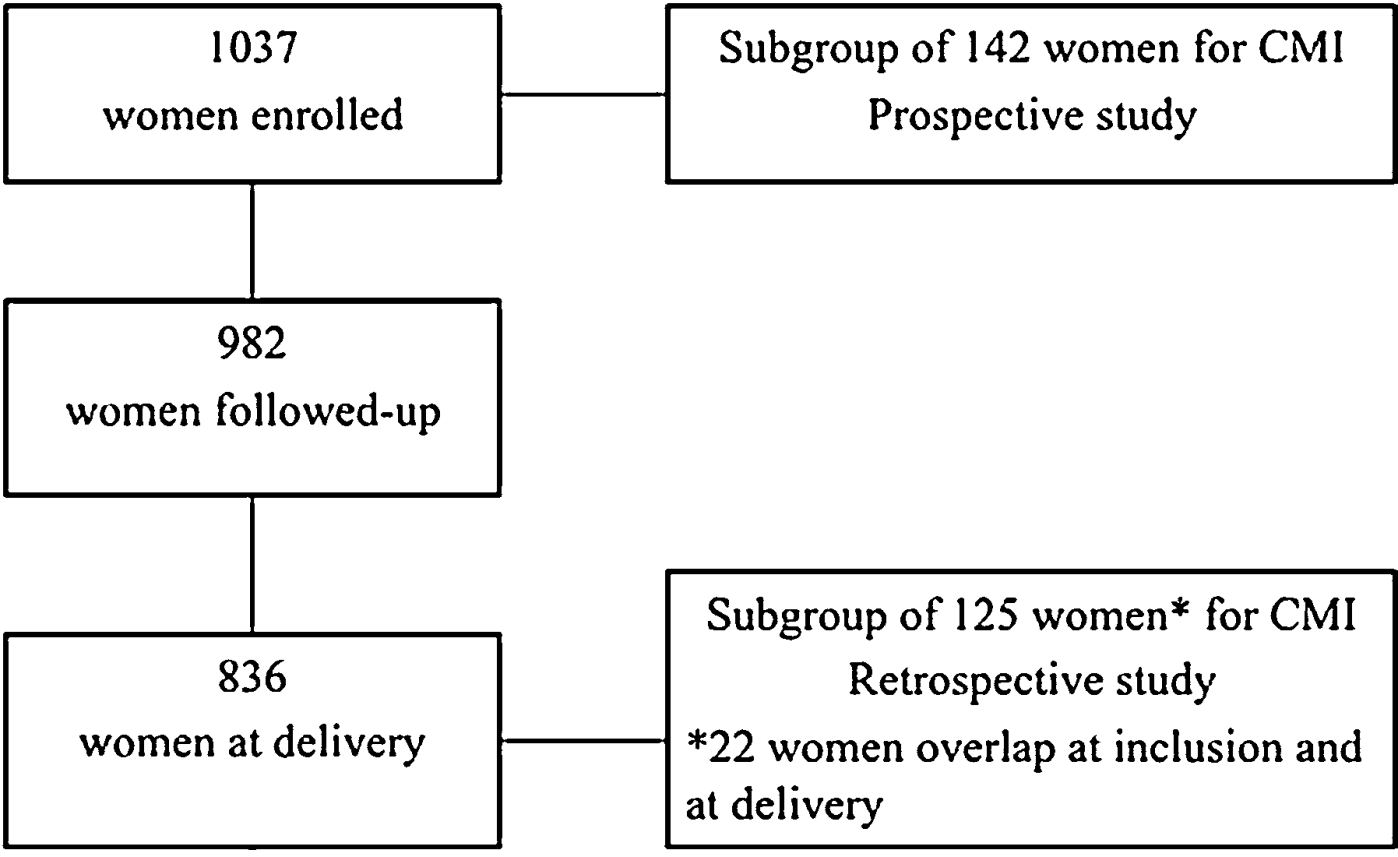
Study design. Within the STOPPAM cohort, the study focused on two specific sub groups in order to investigate: (i) cellular immunological responses early in pregnancy as a predictor of *P. falciparum* infections during pregnancy; (ii) consequences of malaria and anaemia events during pregnancy and at delivery on immune responses at delivery. At inclusion, 142 women were selected harbouring *P. falciparum* infections and a similar number of mothers with no infection. At delivery, 125 women were selected with the malaria history during pregnancy: (i) those who remained uninfected throughout, (ii) those who had had at least one infection during pregnancy but who were not infected at delivery and a third group (iii) who were infected at delivery but who had varying histories of infection during pregnancy

For the study of CMI (cellular mediated immunity) to a recombinant protein corresponding to the DBL-5 domain of the PAM-associated parasite-derived VAR2CSA protein antigen in vitro described here, it was unrealistic to investigate all the women due primarily to cost considerations. The study, therefore, worked only on sub-groups of 142 women at inclusion and 125 at delivery selected within the 1037 women (the target figure was 150 at each time point) (Fig. 1).

1. First at inclusion, women were selected harbouring *P. falciparum* infections and a similar number of mothers with no infection.
2. At delivery, women were selected with the malaria history during pregnancy: (i) those who remained uninfected throughout, (ii) those who had had at least one infection during pregnancy but who were not infected at delivery and (iii) a third group who were infected at delivery but who had varying histories of infection during pregnancy.

Only 22 women were represented in both the inclusion and delivery sub-groups.

### Blood collection, peripheral blood mononuclear cell (PBMC) preparation

Peripheral venous blood samples (10 ml) were collected at different times (inclusion and/or delivery) in tubes containing citrate phosphate dextrose adenine (CPDA). All samples were transported within 4 h to the laboratory of the research centre for malaria during pregnancy and infancy (CERPAGE). PBMC were immediately isolated by density gradient centrifugation on Ficoll (Fisher Scientific, UK) and freshly isolated cells were used for immunological assays.

### *Plasmodium falciparum* infection status

Clinical and parasitological data were collected from mothers at each ANV and also at delivery. Parasitological status with respect to *P. falciparum* infection was determined using a RDT (Parascreen; Zephyr Biomedical Systems), as well as through routine microscopical examination of thick blood smears (TBS) and quantitative PCR (qPCR) on dried blood spots. Briefly, TBS were prepared, stained with Giemsa and examined by two experienced technicians for the presence and density of parasites. qPCR was performed after DNA extraction from blood spots dried on filter paper using the Chelex method, as described elsewhere [20]. At delivery, TBS were made from both peripheral and placental blood samples, such that maternal infection status at delivery was defined by the presence of parasites in either the placental and/or maternal peripheral blood.

Infection with *P. falciparum* was defined as a positive RDT and/or a positive TBS. Women with a positive PCR result but a negative blood smear and negative RDT result constituted a separate group identified as having sub-microscopic infections (SMI).

### PBMC stimulation and cytokine quantification

3 × 10^5^ PBMC were cultured in 5 ml round-bottomed tubes (BD Biosciences) in 300 µl of complete medium (RPMI, 10 % FCS, 1 % l-glutamine, 50 µg/ml gentamycin; Lonza) to which costimulatory receptors CD28/CD49d (2 µl/ml, BD Biosciences, Grenoble, France) were added or were either left unstimulated or stimulated either with mitogen (PHA 5 µg/ml, Sigma, France) or VAR2CSA DBL-5 antigen (5 µg/ml, a gift from Ali Salanti, was cloned into Baculovirus Centre for Medical Parasitology, University of Copenhagen), as described [21]. Cell cultures were incubated for 20 h at 37 °C in an atmosphere containing 5 % CO_2_. Cell-free culture supernatants (150 µl) were collected after centrifugation of the tubes (at 450 g for 8 min) and subsequently stored in 50-µl aliquots at −80 °C prior to thawing for cytokine measurements.

IL-10, Il-13, IL-17, interferon gamma (IFN-γ), and TNF were quantified in culture supernatants using a commercially available cytometric bead array (CBA soluble protein Flex set assay; BD Biosciences, Grenoble, France), conducted according to manufacturer’s instructions on a FACSCalibur 4-colour cytometer. The assay sensitivity was 0.13 pg/ml for IL-10, 0.6 pg/ml for IL-13, 0.3 pg/ml for IL-17, 0.8 pg/ml for IFN-γ, and 1.2 pg/ml for TNF. When the cytokine concentration in a sample was below the detection limit of the test, an arbitrary value was assigned that corresponded to half of the sensitivity value for the specific cytokine concerned. Results were formatted using the BD CBA analysis software (FCAP array). The choice of these cytokines relies on their role in malaria immunity. The choice of these cytokines relies on their role in malaria immunity and the fact that the authors wished to target, as far as possible, cytokines produced exclusively or primarily by T cells. Placental malaria is associated with increased frequency of IFN-γ and TNF producing T lymphocytes suggesting an important role of those cells and cytokines in protection [22]. The concentrations of the IL-10, TNF, and IFN-γ have been shown to be increased in placental or peripheral plasma in women with PAM [9, 23, 24].

### Frequencies of IFN-γ-secreting T cells assessed by flow cytometry

1 × 10^6^ PBMC were cultured in 1 ml of medium under the conditions described above except that, after 12 h of stimulation, secretion of proteins was inhibited by the addition of GolgiStop reagent (1 µl for 1 ml of culture medium). After 20 h’ incubation at 37 °C in 5 % CO_2_, cells were spin-washed with PBS-3 % FCS at 450 g for 8 min. IFN-γ intra-cellular staining was performed according to manufacturer’s recommendations (BD Biosciences), all incubation steps being done in the dark. Briefly, 5 µl of FcR blocking reagent (Miltenyi Biotec, Cologne, Germany) were added to cells from each culture condition. After incubating the cells for 15 min at 4 °C they were labelled by adding 5 µl of anti-CD8-FITC (BD Biosciences), and 5 µl of anti-CD4-PerCP (BD Biosciences). Cells were lightly vortexed and incubated for a further 30 min at 4 °C. After spin-washing once at 450 g for 8 min with PBS-3 % FCS, the cells were resuspended in Perm-Wash Buffer (BD Biosciences) for 20 min at 4 °C. After again spin-washing with Perm-Wash Buffer, 3 µl anti-IFN-γ-PE (BD Biosciences) were added and cells incubated at 4 °C for 30 min. Finally, cells were spin-washed again with Perm-Wash Buffer and acquired on the flow cytometer (BD FacsCalibur).

### Statistical analysis

The associations between maternal characteristics or pregnancy outcomes and cytokine responses or frequencies of T cell secreting IFN-γ, using univariate followed by multivariate linear regression, using robust variance estimation were investigated. The analyses were thus performed in two steps: first a univariate model which aimed to identify potential confounders and the crude associations between cytokine responses and the pregnancy outcomes using the non-parametric Kruskal–Wallis and Mann–Whitney tests. In a second step, association leading to a p value less than 0.2 were selected for the following multivariate model. The baseline characteristics subsequently selected for inclusion in the multivariate analyses were gravidity, maternal anemia (Hb < 11 g/dl), premature birth (<37 weeks) and *P. falciparum* infection (microscopic and sub-microscopic).

Following the hypothesis, the profile of DBL-5-specific cellular immunological responses observed at delivery should reflect exposure to *P. falciparum* infection during pregnancy, but that the presence of infection at delivery could alter or mask such profiles. The detailed infection histories that were collected from STOPPAM mothers via the close surveillance conducted between inclusion into the study and delivery benefit to better elucidate these issues. Those histories allowed to split the delivery sub-group in three groups of women: (i) those who remained uninfected throughout; (ii) those who had had at least one infection during pregnancy but who were not infected at delivery; and a third group, and (iii) who were infected at delivery but had varying histories of infection during pregnancy.

Statistical significance in all multivariate analyses was considered if *p* values were <0.05. All analyses were performed using the Stata/MP 12.0 (StataCorp, College Station, TX, USA) and graphs were made with GraphPad (Prism 6.0).

## Results

### Characteristics of the study population

Table 1 gives an overview of the sub-groups’ characteristics. In terms of their gravidity, their age distribution, as well as of the proportions of low birth weight (LBW) babies and of anaemic mothers, the sub-groups displayed broadly similar profiles. Since the presence of infection with *P. falciparum* was a criterion for entry into the sub-groups both at inclusion and at delivery, it is according to expectation that those parameters differ markedly between the sub-groups. In the sub-group selected at inclusion, 50 % were infected according to the results of examination of TBS whilst 19 % of the same individuals were found to be infected at delivery. In the sub-group selected at delivery, 30 % were infected according to TBS whilst 14 % of the same individuals were found to be infected at inclusion. PCR-based detection consistently augmented the proportions found to be infected regardless of the sub-group or the time-point concerned. For reference purposes, one can note that in the whole STOPPAM cohort infections were detected by microscopy (of TBS) and PCR in, respectively, 16 and 40 % mothers at inclusion, compared with 11 and 34 % at delivery [4]. All microscope-positive TBS were also PCR positive.

**Table 1:** Study population characteristics

	Inclusion sub-group (n = 142)^a^	Delivery sub-group (n = 125)^a^
Primigravid	38	23
Secundi-/multi-gravid	104	102
Age, mean (SD) in years	25 (6)	26 (6)
Gravidity score, mean (SD)	2.9 (1.9)	3.2 (1.9)
% Low birth weight (LBW: <2500 g)	13	15
% Anemic (Hb < 11 g/dl)		
At inclusion	70	60
At delivery	42	41
At inclusion		
*P. falciparum* infected, n (%)		
Microscopy	71 (50)	25 (14)
PCR	19 (13)	34 (25)
At delivery		
*P. falciparum* infected, n (%)		
Microscopy	20 (19)	37 (30)
PCR	36 (20)	37 (30)

^a^22 women overlap at inclusion and at delivery

### Cytokine responses of PBMC at inclusion and at delivery

PBMC from the two different sub-groups of pregnant women at inclusion and at delivery were stimulated with mitogen (PHA) or with the recombinant protein DBL-5. The cytokines IL-10, IL-13, IL-17, IFN-γ, and TNF were quantified in culture supernatants. All data are presented after subtraction of cytokine concentrations in the supernatants of unstimulated PBMC from those in supernatants of stimulated cells (Table 2). Notably, IL-17 was present at low levels after mitogen stimulation but was undetectable in unstimulated and DBL-5-stimulated samples. The other cytokines quantified were present, as expected, at appreciable levels following mitogen stimulation, whilst DBL-5 stimulated appreciable production of both IL-10 and TNF, but comparatively little IFN-γ or IL-13. Regardless of the mitogen stimulation, the concentrations of IFN-γ, IL-10, IL-13 and TNF and only for TNF after DBL-5 stimulation were consistently lower in supernatants of cultures of PBMC taken at delivery compared to those of PBMC taken at inclusion (Table 2; see Additional file 1). A similar pattern was evident with respect to the frequencies of T cells secreting IFN-γ as detected by intracellular staining (Table 3). Thus, regardless of cell type (CD4^+^ or CD8^+^) the frequencies of T cells producing IFN-γ following mitogen stimulation of PBMC at delivery were significantly lower than the frequencies observed at inclusion (univariate analysis p = 0.0013, p = 0.0002 respectively), whilst the frequencies detected following stimulation with the parasite-specific DBL-5 protein were similar at the two time-points (Table 3).

**Table 2:** Cytokine concentrations in culture supernatants following in vitro stimulation of PBMC

	IL-10	IFN-γ	TNF	IL-13
Inclusion (n = 130)^a^				
PHA	643 (216–1291)	4807 (1759–9021)	8567 (5081–14,620)	684 (291–1205)
DBL-5	341 (0.06–847)	0.4 (0.4–259)	3710 (1643–6872)	0.3 (0.3–13)
Delivery (n = 109)^a^				
PHA	233 (0.06–1261)^b^	1303 (332–4326)^b^	6279 (3404–9470)^b^	364 (65–757)^b^
DBL-5	195 (0.06–712)	0.4 (0.4–0.4)	2568 (1521–4981)^b^	0.3 (0.3–0.3)

Values are median concentrations (IQR) in pg/ml

^a^Numbers are reduced due to insufficient quantities of PBMC to perform all assays

^b^Cytokine concentration significantly lower than corresponding concentration in supernatants of PBMC at inclusion (p < 0.05 in all cases)

**Table 3:** The frequencies of IFN- γ -secreting cells following in vitro stimulation of PBMC

	% IFN-γ secreting CD4^+^ T cells	% IFN-γ secreting CD8^+^ T cells
Inclusion (n = 92)^a^		
PHA	6.95 (4.65–9.74)	6.37 (4.02–8.59)
DBL-5	1.44 (0.49–2.32)	1.71 (0.67–2.46)
Delivery (n = 61)^a^		
PHA	4.28 (2.85–7.08)^b^	3.34 (2.01–5.49)^b^
DBL-5	1.47 (0.65–2.02)	1.49 (0.98–2.27)

Values are median frequencies (IQR)

^a^Numbers are reduced due to insufficient quantities of PBMC to perform all assays

^b^Frequencies significantly lower than corresponding frequencies at inclusion (p < 0.001 in both cases)

### The influence of maternal anaemia and gravidity on the cytokine responses of PBMC at inclusion and delivery

**Fig. 2:**
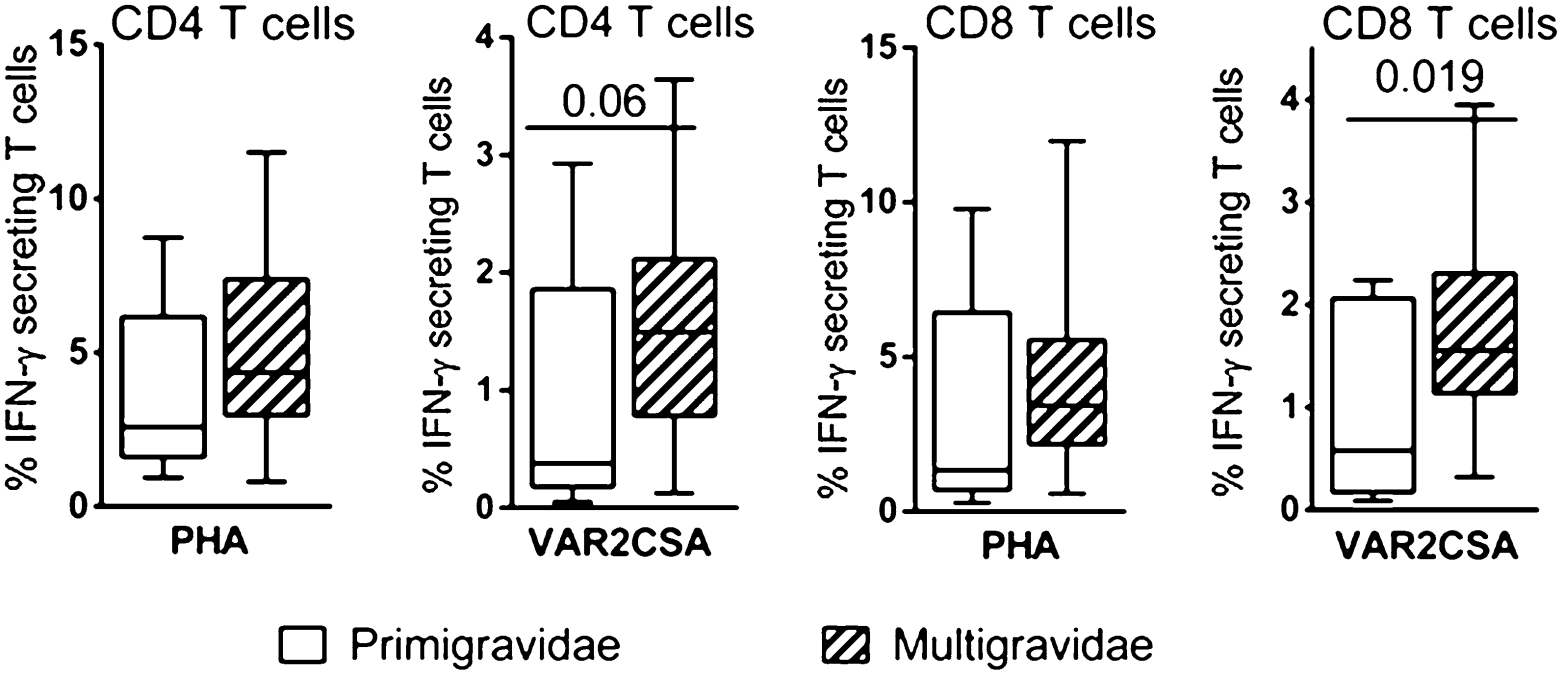
Cytokine responses in PBMC after specific and non-specific stimulation at delivery according to gravidity. IFN-γ-secreting T cells frequencies in response to PHA and to recombinant protein DBL-5 compared primigravidae women with multigravidae women (≥two pregnancies). All *box plots* illustrate medians with 25th and 75th percentiles and whiskers for 10th and 90th percentiles. p values were determined by the non-parametric Mann–Whitney U test in an univariate analysis and significant differences are depicted by p < 0.05

Multivariate analyses showed anaemia at delivery to be associated with a significantly higher TNF response to PHA (p = 0.01) at delivery, but no other associations were evident. Univariate analyses (Fig. 2), comparing PBMC at delivery of primigravidae with those of secundi-/multi-gravidae women (≥two pregnancies), revealed higher frequencies of DBL-5-specific IFN-γ-producing CD4^+^ and CD8^+^ T-cells (p = 0.06 and p = 0.019, respectively) in multigravid compared to primigravid women at delivery. Multivariate analyses controlling for maternal anaemia, premature birth and *P. falciparum* infection did not reveal significant associations with gravidity.

### The influence of current *P. falciparum* infections either at inclusion or at delivery on cytokine responses of PBMC on both inclusion and delivery sub-groups respectively

Since there was no a priori knowledge of the possible effects of sub-microscopic *P. falciparum* infections (those identified solely by PCR) on the cellular immunological parameters were assessed, it was important to first determine whether the differences seen in those parameters were detectable as a function of the presence of such infections or not [4]. In univariate analyses comparing either cytokine concentrations in supernatants or the frequencies of IFN-γ-producing T-cells, for both stimulus (PHA/DBL-5) and at either time-point, there were no discernible differences or marked trends that distinguished the groups with sub-microscopic infections from those either with microscopic or with no infections (data not shown). Nevertheless, for subsequent multivariate analyses those identified solely by PCR as being infected, i.e., truly sub-microscopic infections were considered separately as a group (SMI) for comparison of their cytokine responses with those of the microscopically infected (MI) and the uninfected (UI) groups of women.

Univariate analyses of data from the inclusion sub-group revealed a significantly increased amount of mitogen-induced TNF from PBMC of the MI compared with the UI group, but this was the only difference observed between the groups UI versus MI versus SMI. For the delivery sub-group, infection at delivery was associated with a significantly diminished level of IL-10 in response to both PHA and DBL-5, as well as a significantly reduced frequency of DBL5-specific IFN-γ-producing CD4^+^ T cells in MI versus UI. Multivariate analyses controlling for relevant confounders confirmed those observations concerning the differing profiles of production of TNF at inclusion and of IL-10 at delivery in PBMC of the MI group.

### The influence of *Plasmodium falciparum* infections during pregnancy and/or at delivery on cytokine responses of PBMC from the delivery sub-group

**Fig. 3:**
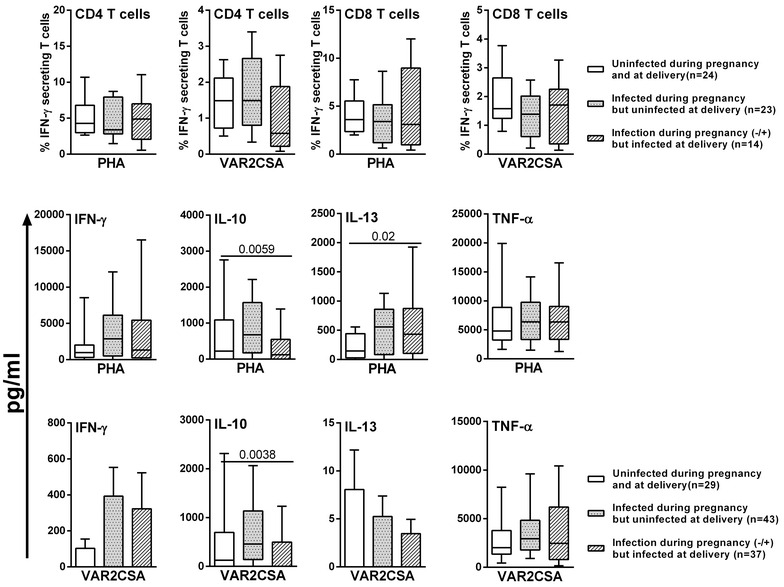
Cytokine responses in PBMC at delivery according to malaria histories during pregnancy. The frequencies of IFN-γ-secreting T cells and cytokine production in PBMC at delivery in response to PHA and to recombinant protein DBL-5 are shown. The three groups of women were defined based on their exposure to *P. falciparum* infection during pregnancy: (i) those who remained uninfected throughout (*white box plot*), (ii) those who had had at least one infection during pregnancy but who were not infected at delivery (*grey box plot*), and (iii) who were infected at delivery but who had varying histories of infection during pregnancy (*black*-*hatched box plot*). Except for IFN-γ and IL-13 production in response to DBL-5, all *box plots* illustrate medians with 25th and 75th percentiles and whiskers for 10th and 90th percentiles. The *box plots* for IFN-γ and IL-13 production in response to DBL-5 show the mean with SEM (standard error of the mean). The statistical significance of differences between groups was determined using the non-parametric Kruskall Wallis test, p < 0.05

**Fig. 4:**
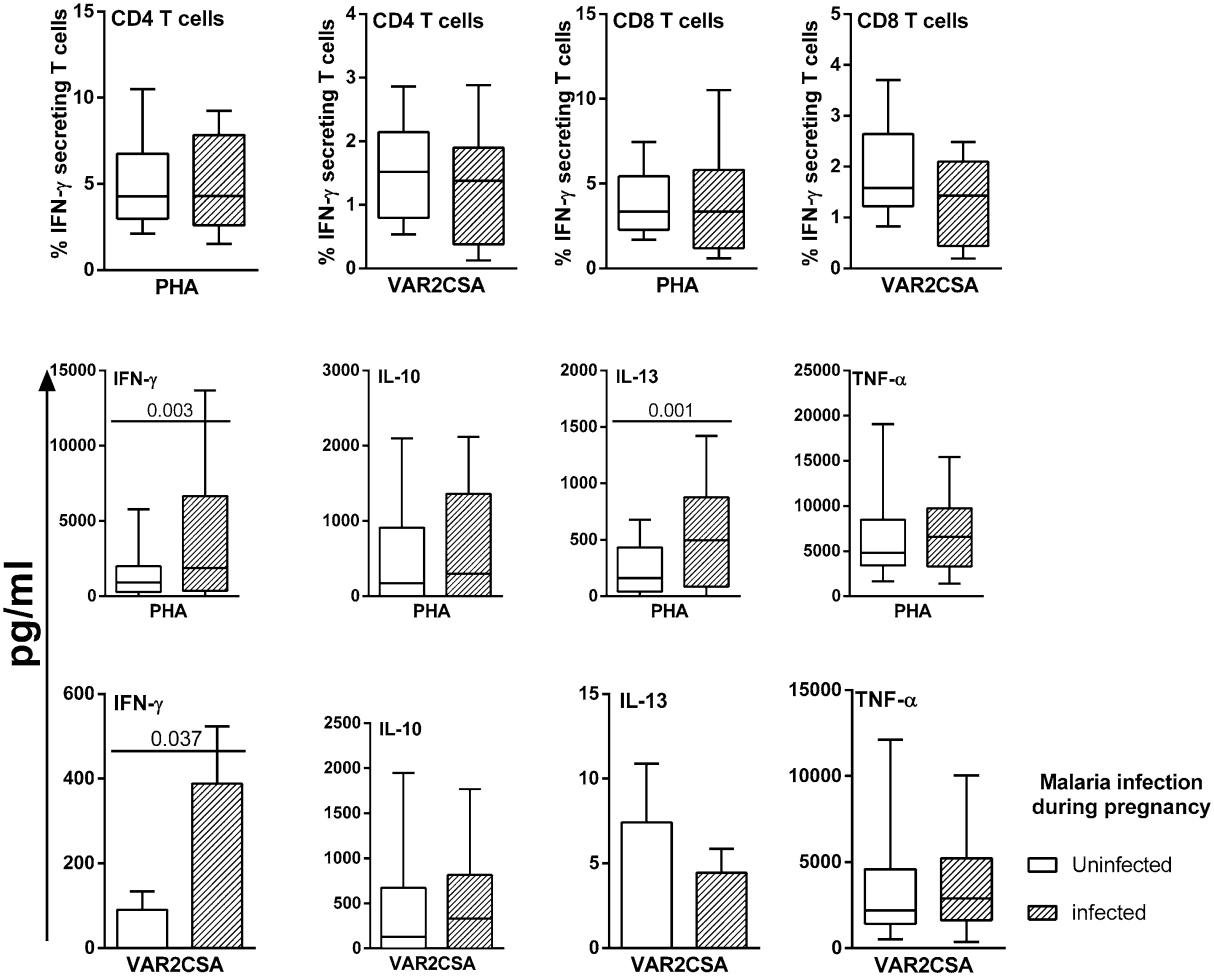
Cytokine responses of PBMC at delivery according to *P. falciparum* infections during pregnancy. The frequencies of IFN-γ secreting CD4^+^ and CD8^+^ T cells and cytokine production in PBMC at delivery in response to PHA or to the recombinant protein DBL-5 are shown. Excluding malaria infections at delivery and according to malaria infection during pregnancy, two groups were defined with uninfected women (*white*) and infected women (*black*). Excepted for IFN-γ and IL-13 production in response to DBL-5, all *box plots* illustrate medians with 25th and 75th percentiles and whiskers for 10th and 90th percentiles. The *box plots* for IFN-γ and IL-13 production in response to DBL-5 indicate the mean with SEM (standard error of the mean). The statistical significance was determined using multivariate analysis model, p < 0.05

The profile of DBL-5-specific cellular immunological responses observed at delivery was predicted to reflect exposure to *P. falciparum* infection during pregnancy, but the presence of infection at delivery could alter or mask such profiles. In order to better elucidate these issues use was made of the detailed infection histories that were collected from STOPPAM mothers via the close surveillance conducted between inclusion into the study and delivery. Those histories allowed identification of three groups of women described in the methods, (i) those who remained uninfected throughout, (ii) those who had had at least one infection during pregnancy but who were not infected at delivery and a third group (iii) who were infected at delivery but who had varying histories of infection during pregnancy. Figure 3 illustrates the cytokine responses of these three groups reflecting exposure or not to *P. falciparum* infection during pregnancy, revealing that infection at delivery is associated with a reduction in IL-10 responses both to mitogen and to DBL-5. Multivariate analyses showed that PBMC at delivery of mothers with a history of infection during pregnancy but who were uninfected at delivery displayed significantly increased production of IFN-γ in response to both PHA and DBL-5 stimulation (p = 0.003 and p = 0.037, respectively) as well as increased PHA-specific IL-13 (p = 0.001) compared to PBMC of uninfected women (Fig. 4).

Separately, the relationships between the cumulated number of *P. falciparum* infections during pregnancy and the cytokine profile displayed by PBMC at delivery were also examined, segregating women into three groups: (i) uninfected during pregnancy, (ii) one *P. falciparum* infection during pregnancy, and (iii) two or more *P. falciparum* infections during pregnancy, excluding those with infections at delivery from these analyses. Multivariate analyses that adjusted for potential confounders (anaemia, gravidity, premature birth), revealed significantly higher levels of IFN-γ (p = 0.004) and IL-13 (p = 0.019) in response to PHA and a significantly higher level of IFN-γ (p = 0.04) in response to DBL-5 of PBMC from mothers more often infected during pregnancy compared to uninfected mothers (data not shown). These analyses also revealed a lower frequency of DBL-5-specific IFN-γ-secreting CD8^+^ T cells (p = 0.022) as a function of increasing numbers of *P. falciparum* infections during pregnancy.

### Cellular immunological responses at inclusion as a predictor of *Plasmodium falciparum* infections during pregnancy

Whether the cytokine responses or frequencies of IFN-γ-secreting T cells upon in vitro stimulation at inclusion were predictive of *P. falciparum* infections during pregnancy were determined. Multivariate analysis revealed independent associations for an increased risk of *P. falciparum* infection during pregnancy with (i) higher TNF, and (ii) a higher frequency of IFN-γ secreting CD8^+^ T cells, both in response to PHA (p = 0.04, p = 0.051, respectively), at enrolment.

## Discussion

This study was designed to generate information to allow both prospective and retrospective analyses of the interactions between cellular immunological responses and infections with *P. falciparum* during pregnancy. The prospective analyses revealed two main findings concerning, on the one hand, non-specific CD8^+^ T-cell IFN-γ responses, and on the other, non-specific TNF responses. In the first case, women more likely to be re-infected during pregnancy were those in whom an elevated non-specific CD8^+^ T-cell IFN-γ response was detectable at inclusion. Notably here is the fact that, in retrospective analyses, antigen-specific CD8^+^ T-cell IFN-γ responses declined as a function of increasing numbers of re-infections. These findings could point to CD8^+^ T cell IFN-γ responses as being detrimental in the context of protection from *P. falciparum* infections during pregnancy. These observations in this context are novel and merit further investigation. Asexual blood stage parasite-antigen specific CD8^+^ T-cell responses are a documented feature of controlled human malaria infections in adults [25]. The function, if any, of such cells remains a paradox given that their potential targets, erythrocytes, do not express HLA Class I molecules. Based on studies in a mouse malaria model it is conjectured that they may have a ‘bystander’ role concerning activation of phagocytic activity [26].

The second finding from prospective analyses concerned elevated non-specific TNF responses at inclusion that were a prognostic marker for subsequent infections. This observation is clearly incompatible with the purported protective role against infection detected at delivery attributed to non-specific proinflammatory responses measured at recruitment in a cohort of Papua New Guinean women [27]. Whether or not, in the context of the latter study, (i) the timing of recruitment and/or (ii) lifelong exposure to both *P. vivax* and *P. falciparum* have any bearing on this issue remain open questions. The prospective analyses in that study, it should nevertheless be noted, did also show that lower non-specific Treg-IL-10 (anti-inflammatory) levels were associated with future *P. falciparum* infections. Taken on its own, that observation is not incompatible with a higher non-specific proinflammatory (e.g. TNF) response being associated with future infection, as was observed here. In all this, it seems to us clear that the issue of the quality of the cellular immunological response involved is likely to have as much importance as its quantity. Thus, a proinflammatory response generated via an antigen-specific interaction that is guided predominantly by IFN-γ will almost certainly differ in quality from a proinflammatory response generated via a non-specific route that is guided predominantly by TNF. It is perhaps also instructive to note that in our own and others’ studies the peripheral plasma levels of neither IFN-γ nor TNF vary as a function of the presence of *P. falciparum* infections during pregnancy but that their placental plasma levels—measured at delivery—certainly do [28–30]. For obvious reasons the placental compartment, other than at delivery, remains uncharted territory for the sorts of studies that could help to answer some of the outstanding immunological questions in the context of an infection during pregnancy that is localised to the placenta itself. This is, then, the major limitation of the study presented here, since assessments of the responsiveness of circulating T cells may represent only an approximation of events localised to and focused on the placental space.

In retrospective analyses, the aim was to assess the impact of *P. falciparum* infections documented during pregnancy on the cellular immunological responses measured at delivery. The findings in this regard point clearly to the generation of an IFN-γ-led antigen-specific T-cell memory response, measurable in peripheral blood samples at delivery, a type of response that is, as the data further show, augmented by re-infections during pregnancy in a manner that does not reflect the gravidity. As mentioned above, a concomitantly reduced antigen-specific CD8^+^ T-cell IFN-γ response at delivery as a function of increasing numbers of infections during pregnancy was observed. A previously published study documented a significant gestational age-related decline in the overall frequency, ex vivo, of CD4^+^ T cells between the second trimester and delivery, accompanied by increased frequencies of CD8^+^ T cells [31]. Those profiles contrast clearly with the in vitro stimulation-derived profiles reported here. The latter suggest that pathogen antigen-specific responsiveness is maintained despite pregnancy-related alterations in lymphocyte populations.

Infection at the time of blood draw modified the cellular immunological responses detectable, and the nature of those modifications, furthermore, reflected the time-point (inclusion versus delivery). The authors noted elevated non-specific TNF at inclusion versus diminished specific/non-specific IL-10 at delivery. These findings are consistent with earlier observations showing altered ex vivo peripheral blood cell profiles on the one hand [32] and altered cytokine levels—primarily increased IL-10—on the other [31], as being associated with infection in these women. It is notable here that infection *per se* tends to generate T-cell-mediated cellular responses, whether specific or non-specific, that favour inflammation. In the context of a successful pregnancy, inflammatory activity needs to be counter-balanced by anti-inflammatory activity [33] which likely explains the elevated circulating plasma IL-10 levels consistently associated with *P. falciparum* infections during pregnancy. In the context of the latter, the data presented here suggest that the primary source of infection-related IL-10 is probably not T cells. Placental cells are the most likely source given that the infections are localized there.

Other than the impossibility of accessing the placental compartment during pregnancy, the study’s shortcomings are primarily related to sub-group sizes that were necessarily limited both by resources and by cell numbers (blood volumes) available. A separate issue concerns the limited number of stimuli used, also again a reflection of resources available.

## Conclusions

The findings presented here represent a first step in documenting cellular immunological responses to the candidate vaccine antigen VAR2CSA and, in particular, how infection with *P. falciparum* during pregnancy modulates such responses. As such these observations contribute important information to the design of future evaluations in the specific context of VAR2CSA-based vaccine trials.

## Additional file

10.1186/s12936-016-1525-x Cytokines profile in response to PHA and to DBL-5 stimulation of PBMC at inclusion and at delivery. The frequencies of IFN-γ-secreting T cells and cytokine production in PBMC at delivery in response to PHA or to the recombinant protein DBL-5 are shown. Except for IFN-γ and IL-13 production in response to DBL-5, all box plots illustrate medians with 25th and 75th percentiles and whiskers for 10th and 90th percentiles. The box plots for IFN-γ and IL-13 production in response to DBL-5 indicate the mean with SEM (standard error of the mean). The univariate statistical significance of differences between groups was determined using the nonparametric Mann–Whitney U test, p < 0.05.

## Abbreviations

ANV: antenatal visit
BD: Becton Dickinson
CBA: cytometric bead array
PBMC: peripheral blood mononuclear cell
CERPAGE: *Centre d’Etude et de Recherche sur le Paludisme Associé à la Grossesse et à l’Enfance*
CPDA: citrate phosphate dextrose adenine
HIV: human immunodeficiency virus
IFN-γ: interferon gamma
IL-6: interleukin-6
IL-10: interleukin-10
IL-13: interleukin
IL-17: interleukin-17
IPTp: intermittent preventive treatment during pregnancy
LLIN: long-lasting, insecticidal bed net
LBW: low birth weight
MI: microscopic infection
PBMC: peripheral blood mononuclear cells
IRD: research institute for development
MI: microscopic infection
PHA: phytohemagglutinin
PAM: pregnancy-associated malaria)
RDT: rapid diagnostic test
SMI: sub-microscopic infection
SP: sulfadoxine-pyrimethamine
STOPPAM: strategies to prevent pregnancy associated malaria
TBS: thick blood smear
TNF: tumor necrosis factor
UI: universal unit
WHO: World Health Organization
